# Some Observations on the Use of the Lancet

**Published:** 1881-02

**Authors:** Rufus W. Griswold

**Affiliations:** Rocky Hill, Conn.


ARTICLE II.

### SOME OBSERVATIONS ON THE USE OF THE LANCET.

BY RUFUS W. GRISWOLD, M. D., ROCKY HILL, CONN.

The October, 1880, issue of the INDEPENDENT PRACTITIONER, has an article by Prof. Byrd, one of the editors, on the subject of Blood-Letting in Disease, that has in it so much common sense that

I have a fancy to make some comments upon it. My object is less to offer any differing criticism, th [illegible]n to call attention to some of the reasons why the use of the lancet has largely gone by; although there are some points in the article upon which I may be allowed to not quite agree with the writer of it, and especially this: "That the necessity for the use of the lancet is as great at the present time as it ever was in the past, and that the type of disease has undergone no such change as to render the abstraction of blood unnecessary or improper in the successful management of all cases attended with a full tense and quick pulse." An experience of thirty years in practice, backed by the observations of some of my neighbors who have been driving, for twenty years longer than I have, is to the import that there has been such a change in the type of diseases as renders the frequent use of the lancet less important than formerly; but not such an one as justifies that degree of abandonment of it that at present prevails. In this region, at least, there is less of the sthenic type in even inflammatory fevers; there is a more general disposition to take on what is called the typhoid form; depletion, whether by the evacuation of blood or by the administration of reducing drugs, is not so beneficial towards a recovery from a disease of even the highly inflammatory forms, as formerly; and the legitimate deduction is, that the use of the lancet is less often needed. Certainly, this is the view entertained in the matter by nearly all the practitioners who began work forty and fifty years ago, with whom it has happened to be my pleasure to converse on the subject; and this view is supported by abundant written authority on the subject. I am, therefore, of the opinion that the view of Dr. Byrd, as expressed in the quotation given, is too strongly drawn; while on the other hand, his position, that the non-use of the lancet to the degree now prevalent, is unfortunate, is correct. A majority of the practitioners of the "old school" at the present day will agree with Prof. Byrd in his position on the last point indicated, so far as their *opinions* are concerned, but as a matter of *practice* they follow the fashion of the generation, and do not bleed. The principal object of this paper is to say something about *why* they do not. And first—one reason why they do not, is to be found in the fact of a change in diathesis, as I have briefly indicated. There is much of truth in the common observation of the older men in the profession, that their patients do not bear bloodletting so well as they formerly did. Having discovered this, common sense required that they should bleed less. But this change of diathesis is only one of the factors in the account, and but a minor one. The very general opinion that the physicians of the early half of the present century, and for some time before that, bled too often, is beyond much doubt correct. The doctrine of the purely symptomatic nature of fever, championed by the active and energetic mind of Broussais, in the first quarter of the century, and which became very generally received on the continent of Europe and in America, gave an unfortunate impetus to the use of the lancet, and led to its great abuse. Not only fevers of acknowledged inflammatory character, such as acute pleurisy, etc., were bled; but so, also, were the cases of typhus, typhoid, etc., upon the ground that the fever connected with the cases was of an inflammatory type, and was to be subdued by antiphlogistic remedies.

The idea of the essentiality of the fever became, to a great extent, lost sight of, and the practitioner treated an inflammation and not a fever. Without abandoning the theory into which they had been educated, many physicians began to discover that in certain epidemics of fever a greater percentage of cases were lost after venesection than in a like number of patients under other similar circumstances who were not bled. Reference to the medical literature of the first half of the century shows a great deal of hot discussion between the blood-letters and anti-blood-letters. Out of the observation made and the discoveries engendered there was cultivated a prejudice, not merely professional, but popular, against bleeding, per se, and without reference to conditions, that carried the usage from its abuse to the opposite extreme of abandonment. It is a very general law in all matters, that the farther the pendulum swings to the left the farther will it swing to the right on its return. The pendulum of venesection had swung too far in one direction for the best degree of equilibrium in the treatment of disease, and the return has carried it quite beyond the best degree of equilibrium in the opposite direction. Besides this natural recoil, inevitable and to be expected, there has been added a powerful pressure of an erroneous and illegitimate nature, that has pushed the lancet into a general disuse, quite as unfortunate as its former abuse; and against which other sensible papers like the one of Prof. Byrd, are demanded as stimulants to its partial restoration.

Contemporaneously with the animated discussion upon the advisability of bleeding in certain fevers, carried on by members of the profession, and perhaps in part, out of that discussion, there arose in various sections an illegitimate class of practitioners, mostly illiterate, without professional status and destitute of preliminary medical culture, interchangably known as Botanics, Thompsonians, Eclectics, etc., whose chief stock in trade for public acceptance, in lack of theory from the schools or from standard medical books, and in lack also of knowledge from clinical experience, was denunciation without regard to the conditions encountered, of the use of calomel, antimony, blistering, leeching and bleeding. This denunciation found ready public credit; not only from the mouths of this class, but in various other ways, the doctrine was widely diffused. It is one of the irregularities of medical literature, that outside of the libraries of the profession, you seldom encounter a standard medical work. This class of books is written and published for the profession, and is very seldom, indeed, used by the public at large; but in many sections of country, for the past fifty years, a canvas of the families would show an abundance of works, published for family reading, distributed through the region by agents and peddlers, emanating from irregular practitioners, and all of them saturated with abuse of the methods and modes of treatment of regular physicians. These books have been, and are, loaned from house to house much as the weekly or dime novel; and one result is the indoctrination of the belief that bleeding is not merely harmless, but is pernicious and frought with death. Co-ordinating with this means of false instruction has been and is, the public press, secular and religious, and particularly the religious. The pecuniary interest of the public press in relation to public health, so far as means and methods for the cure of diseases is concerned, is identical with the pecuniary interest of advertising quacks. The public press lends, or rather, sells itself to the diffusion of the ways and means of medical quackery in all its protean forms. The subsidies of traveling impostors and of patent medicine men, fill one of the largest arteries of support for the newspaper print of all degrees and kinds, and the influence upon the public mind of the smallest member of the press, no matter how weak or imbecile may be the intellect that bestrides its tripod, is more potent than the teachings of a hundred of the ablest minds that adorn the profession of medicine. Through this channel the general reader is constantly guarded against those salient methods of treatment in disease—bleeding, blistering, etc., by daily denunciations and warning, as a prelude to the introduction of the methods of the advertisers. Added to this is the influence of itinerant lecturers. Seldom, indeed, is it that an educated and experienced physician takes to the public platform for the purpose of diffusing knowledge in the arenas embraced in the rational practice of medicine. But there is a standing army always engaged in lecturing before the gaping crowd, upon physiology, hygiene, and the likes; and this army is composed of unprincipled pretenders and illy educated or illiterate doctors, without legal or professional status; and one of the chief burdens of their absurd mouthings is animadversion of the modes of practice and methods of procedure of the physicians of the schools. The deeper the illiteracy, the more profound the ignorance of the travelling mountebank, the more sweeping will be his denunciation of the practice of the class of men whose knowledge is based upon the accumulated and recorded experience of acute observers for two thousand years. To him the fathers of medicine were barbarians, and their successors are the deluded followers of an exploded system, continued only by fools. The result of the false teaching of the class of books alluded to and of the medical advertising through the press and of the schooling of the tramping lecturers upon medicine, is, the mass of the community, in many sections, have been seduced to believe that blood letting is a crime against health and a hindrance in recovering from disease, no matter what may be the condition of the case in which its advisability is considered. The average intelligence of even well educated communities goes no farther than to grasp the plausible but fallacious teaching that is thrust every day before it; it does not stop to analyze the motives nor to criticise the arguments in the case, nor indeed is it cultured in this direction to the capacity of justly weighing them at their true significance and value. Therefore, it is the condition obtaining in relation to bleeding, not so much that the sensible physician has lost sight of its value in many cases of disease, as that the community in which he practices will not allow him to take blood.

Public prejudice overrides professional advice, unless the advice runs current with the prejudice, and very little, or nothing, is gained against the prejudice by a recovery after bleeding, since the popular verdict is that the patient would have got well quicker and better without it; an argument that can seldom be disproved. In the same line, in any given case, where venesection has been resorted to, and the patient does not well, the get opponents of the operation triumphantly assert that the bleeding caused death, and that in the absence of it the patient would have recovered, which, also is difficult to disprove. The whole art of the practice of medicine is involved in so many uncertainties as to the results that are to follow the administration of drugs, or the institution of any procedure, however simple, that it may puzzle the most sagacious to determine the weight of any factor introduced, whether for good or for ill. It need not surprise us, therefore, that to minds quite unacquainted with the therapeutical effects of blood-letting in disease, a death that follows a bleeding, however remote in point of time, should be credited to the operation rather than to the disease for which the operation was performed. An uncertain percentage of cases of any acute inflammation will recover, whether bad or not: an uncertain percentage of them will die in spite of the bleeding, and it will not unfrequently happen that of two cases of the same disease one that is not bled will get well and the one that is will succumb. As a matter of fact, it is the case in many communities, that if a patient is bled and then dies, nine of every ten persons in the neighborhood will assert and part of them will believe that the bleeding was an accessory, if not the chief cause of the untoward event, and it is usually quite impossible for the practitioner to show that the nine are not right in their view of the matter.

Under these circumstances it can hardly surprise us that the use of the lancet has gone out of fashion. It is not so much that we have less faith in its benificence, rationally employed, as that our patients are opposed to it. Whether, in spite of that opposition, we should employ it oftener than we do is a question that every one must answer for himself. It may be possible for a bold and determined man to work up that road to confidence with his patients, but the path is at present so beset with difficulties that a hundred will fail by the way that one succeeds. A single death, after phlebotomy, will do more to impede the success of a young man in the profession than a dozen deaths without it; it is wise, therefore, to be cautious in the use of so potent a remedy, and to sin less in commission than omission of opening a vein. It may be said that whether he succeeds or fails, it is the duty of the physician to do in all cases what he thinks will be the best for his patient. The position may have its merit, but on the other hand, there is no law of right that demands of a practitioner that he shall assume the responsibility of all the ignorance and prejudice in which his patients have allowed themselves to be educated.

Rocky Hill, December, 1880.

